# Patient perceptions of three-dimensional (3D) surface imaging technology and traditional methods used to assess anthropometry

**DOI:** 10.1016/j.obpill.2024.100100

**Published:** 2024-02-01

**Authors:** Lucie Nield, Michael Thelwell, Audrey Chan, Simon Choppin, Steven Marshall

**Affiliations:** a Advanced Wellbeing Research Centre, Sheffield Hallam University, Olympic Legacy Park, Sheffield, S9 3TU, UK; b Sheffield Business School, City Campus, Sheffield Hallam University, S1 1WB, UK

**Keywords:** Body morphology, Weight management, Body image, 3D surface imaging, Obesity

## Abstract

**Background:**

Obesity and overweight are commonplace, yet attrition rates in weight management clinics are high. Traditional methods of body measurement may be a deterrent due to invasive and time-consuming measurements and negative experiences of how data are presented back to individuals. Emerging new technologies, such as three-dimensional (3D) surface imaging technology, might provide a suitable alternative. This study aimed to understand acceptability of traditional and 3D surface imaging-based body measures, and whether perceptions differ between population groups.

**Methods:**

This study used a questionnaire to explore body image, body measurement and shape, followed by a qualitative semi-structured interview and first-hand experience of traditional and 3D surface imaging-based body measures.

**Results:**

49 participants responded to the questionnaire and 26 participants attended for the body measurements and interview over a 2-month period. There were 3 main themes from the qualitative data 1) Use of technology, 2) Participant experience, expectations and perceptions and 3) Perceived benefits and uses.

**Conclusion:**

From this study, 3D-surface imaging appeared to be acceptable to patients as a method for anthropometric measurements, which may reduce anxiety and improve attrition rates in some populations. Further work is required to understand the scalability, and the role and implications of these technologies in weight management practice. (University Research Ethics Committee reference number ER41719941).

## Graphical abstract

**Figure:**
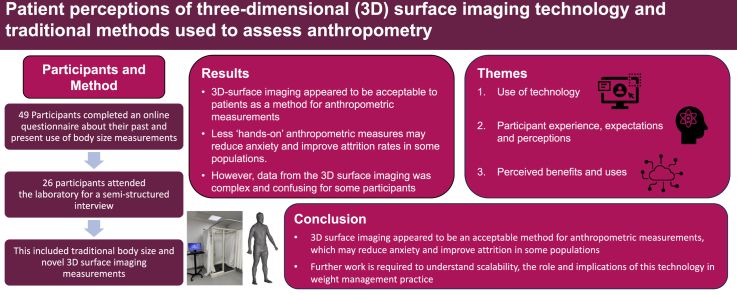

## 1 Introduction/background

Obesity is a widespread, chronic, complex disease associated with excess fat mass, and contributes to many weight related comorbidities such as cancer, heart disease, stroke, depression and premature mortality [1]. Obesity incurs a huge cost both financially, societally, and personally to those living with obesity.

Overweight and obesity are traditionally assessed using the body mass index (BMI) which gives a ratio of a person's weight to their height squared [2]. BMI provides a measure of relative obesity and stratifies individuals into categories (underweight, normal weight, overweight and obese) according to World Health Organization (WHO) guidelines [3,4] Whilst this is important clinically and on a population level, BMI is strongly contested as a useful measure for individuals as it does not accurately reflect variations in body composition, weight distribution, and other features of human morphology [[5], [6], [7], [8]] which can lead to misclassification of individuals [9].

Weight management services traditionally involve a holistic assessment of the individual, including an understanding of their body size and shape, and how this relates to their current weight status. However, many service users can be uncomfortable with being weighed or measured [10] for several reasons, such as invasion of personal space and body measures reflecting an unflattering personal attribute [11]. It is hypothesised that this may deter individuals from attending weight management services or contribute to high attrition rates. In addition, weight stigma is experienced from healthcare professionals (HCPs) and avoiding being weighed is a primary reason why women avoid attending appointments [12]. Reasons given for women refusing to be weighed can range from shame and embarrassment, to concerns about discrimination [12]. Therefore, identifying interventions and alternative ways of measuring the human body that are non-stigmatising could reduce the negative impact of body measurement in healthcare.

Emerging new technologies for assessing human morphology, such as three-dimensional (3D) surface imaging technology, could provide an attractive alternative to traditional manual anthropometric techniques [13]. Modern 3D surface imaging systems, acquire point cloud data that explicitly capture surface topography, which can provide detailed and accurate external dimensions and shape characteristics of the human body, such as curvature and partial volumes [14,15]. Surface features extracted from this 3D imaging data can be used to characterise individuals according to their shape [16], as well as their size, to a higher degree of precision and complexity than existing manual methods [17,18]. Several imaging techniques can be used to create full body geometries, with a range of commercial 3D imaging systems currently available [[19], [20], [21], [22]], each varying in their underlying technologies, cost, functionality and accuracy. Examples of the most common imaging techniques are presented in Table 1.

**Table 1:** Overview of different techniques and commercially available 3D imaging systems for body measurement.

Imaging technique	Brands/Models	System type	Cost (USD$)	Accuracy	Scan duration
Laser line [23,24]	Cyberware WBX Vitrionics Vitus Smart	–	∼37,000–240,000	<2 mm 27 points/cm^3^	∼10–15 s
Stereo Photogrammetry [25,26]	Cranfield Vectra 3dMDbody e.g. Flex8	Passive Hybrid	∼190,000	0.2 mm <0.2 mm	∼2–8 msecs ∼1.5 msecs
Structured light [27,28]	Artec Eva/Spider TC2 KX-16 SizeStream	Blue light Infrared Infrared	∼10,000–20,000	0.1/0.05 mm 1 mm 1 mm	161/8 fps 3 s 6 s
Depth sensor [[29], [30], [31]]	Microsoft Kinect V2 Intel Realsense D435	Time of flight Stereoscopic	∼200/per device (∼1000/system)	<6.5 mm >6.5 mm	<10 s (single camera)/∼0.8 s (multi camera)

Typically, 3D surface imaging techniques utilise some form of projected light to acquire external body shape data - laser line, infrared or blue light – which capture external dimensions of the human body quickly (<10 s), and without the need to touch the individual or expose them to harmful radiation that is associated with imaging modalities such as computed tomography (CT). Though 3D surface imaging technology was previously expensive, increased use of 3D surface imaging in entertainment, fashion, ergonomics, and health has bolstered the market, leading to reduced prices and greater accessibility [32]. Recent literature has suggested that 3D surface imaging technology can be a potential method for estimating body composition [19,[33], [34], [35], [36], [37], [38], [39], [40], [41], [42], [43]] and can provide precise, reliable, accurate [44] and meaningful information about the quantity and distribution of fat throughout the body [45]. Moreover, commercially available 3D body scanning has been shown to provide valid estimations of total body volume and relative body fat mass in comparison to air displacement plethysmography [46] and accurate estimations of fat mass, fat free mass and % body fat when compared to a 4-component model (Dual-energy x-ray absorptiometry) [47]. However, like other anthropometry-based estimation techniques, body composition prediction using 3D imaging is a doubly indirect method relying on the use of regression algorithms [48], which are dependent upon the accuracy of the raw 3D imaging data. An assessment of commercially available scanners highlighted variability in the validity of, and proportional bias for all scanners that were assessed [47]. Acknowledging its limitations, 3D imaging provides the possibility of quick, contactless, ionizing radiation-free body composition measurement without the need for a technician suitable for regular use [34,42]. It is possible that this novel technology, could provide greater granularity to anthropometric and morphological measures which may prevent the of misclassification of overweight and obesity and provide an alternative way to measure changes in weight, which could appeal to some individuals, where physical touch is culturally, practically, or socially unacceptable.

However, attitudes towards this technology as an alternative anthropometric tool for use within healthcare and weight management are currently unclear. The prevalence of individuals that suffer from issues relating to body image and body dysmorphic disorder (BDD) [49] is increasing and has potentially accelerated due to the emergence of social media [50]. Therefore, the need for sensitivity when collecting body measurement data from individuals and presenting their images back to them is paramount, due to the potential risks of causing a negative psychological response pertaining to body image issues.

The aim of this study was to understand acceptability of traditional and 3D surface imaging-based body measures, and whether perceptions of these techniques differ between population groups.

To achieve this aim, we sought to answer the following research questions.

1) What are the current methods and strategies employed by individuals to monitor their body weight and/or composition?
2) What are the key drivers behind the strategies employed to monitor their body weight and/or composition?
3) How does the emotive response differ between established methods of assessing body morphology and does the use of novel 3D surface imaging technology produce a different response?

## 2 Methods

### 2.1 Participants

The primary outcome in this study was subjective perceptions of acceptability towards traditional and novel methods for assessing body shape. The inclusion criteria for this study were that participants needed to be aged 18 years or above and be able to stand unaided for approximately 5 min to enable them to complete the 3D imaging procedure. Participants also needed experience of possible weight loss strategies; however, they did not need to be actively trying to lose weight at the time of the study. Body weight metrics were not an inclusion criterion for this study. Participants were recruited via social media, e-mail, word-of-mouth and posters. Additional face-to-face promotional events were also held in public venues including the city library, shopping centre and university buildings.

Participants were given a detailed participant information sheet before the study commencement, detailing the study protocol and providing information about the voluntary nature of their input, assurance of data anonymisation, and maintenance of confidentiality. Consent was confirmed prior to completing the questionnaire and the on-site interview. Participants were free to withdraw from the study at any point during the process although any data captured up the point of withdrawal would be stored securely and used in an anonymised format. Participants were offered a £20 shopping voucher as a thank you for attending the session. Recruitment methods signposted potential participants to a short online questionnaire via Qualtrics.

Ethical consent was gained via a robust peer-reviewed University research ethics process at Sheffield Hallam University (REC number ER41719941).

### 2.2 Measures

The questionnaire collated basic demographic information such as age, gender, postcode and education status and also included the Cosmetic Procedure Screening questionnaire (COPS) for BDD [51]. Postcode data allowed researchers to calculate the Index of Multiple Deprivation (IMD) The questionnaire aimed to determine how a person engages with their own self-image, whether and how they track their own body size and shape, and what methods and strategies they currently employ, or have previously employed if trying to alter their own self-image (through weight loss or physical training for example) and to monitor the physical aspects of their body such as shape and size (e.g., weighing scales, tape measure, photographs etc.) (see questionnaire in supplementary materials).

Participants were eligible for the second part of the study if they were over 18 years of age and able to stand unaided for 5 min or more (necessary during 3D surface imaging). Participants should have tried to manage their weight in the past (for any reason) but did not need to be actively trying to lose weight at the time of the study. Respondents who screened positively for body dysmorphia (a screening tool score higher than 40) [51] were not invited to further follow-up interview sessions as the researchers felt it was unethical to subject those at potential risk of living with body dysmorphia to further detailed scrutiny of their body shape and size.

There were 53 responses to the questionnaire, but after removal of duplicates, there were 49 unique respondents. Twenty-six participants completed the follow-up interview and laboratory visit (Sheffield, UK). Participants were asked to attend the clinic with some form-fitting clothes. Cycling shorts and a vest top were provided by the research team if necessary.

The interview was a semi-structured session with two parts which took part face-to-face in the body morphology laboratory at a time that was convenient for the participant. Initially, the interviewer explored themes arising from the initial questionnaire. The interview explored their current and previous use of body measurements, perceptions of ‘future technologies’ for weight management, and gathered initial ideas and impressions of 3D surface imaging as a potential technology (see semi structured interview in supplementary materials).

**Fig. 1:**
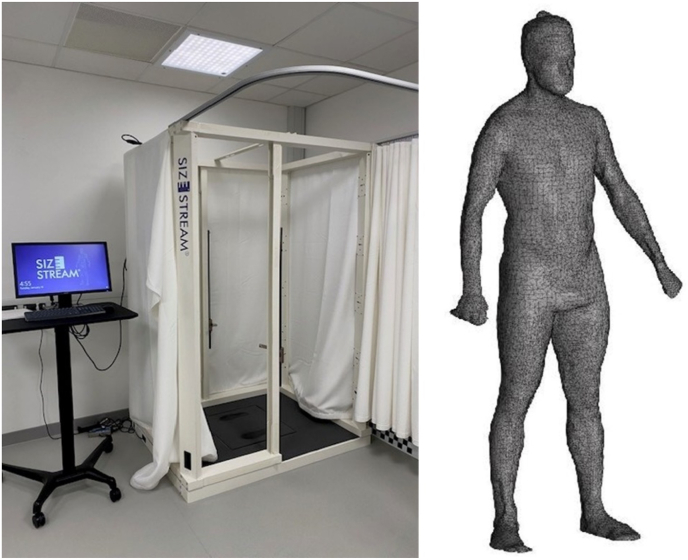
Image of 3D imaging device used and example of extracted data.

After the first stage of the interview each participant had traditional body morphology measures taken, and a 3D surface image. The traditional measures included: a Leicester height stadiometer (Marsden, UK), digital weight scales (Conair, UK), hip and waist girth measures using a basic anthropometric tape measure (Lufkin Executive Thinline 2 m, W606PM). A Size Stream SS20 3D surface imaging device (Size Stream, Cary, NC, USA), was used to obtain 3D surface images of participants (Fig. 1).

Prior to the 3D surface image scan, participants were provided with appropriately sized, form fitting clothing. During the scanning procedure, participants were asked to adopt a standardised pose, as demonstrated in the example of extracted data in Fig. 1, and stand still for approximately 10 s.

Data was shared with all participants at the interview, and a standard 3D model was shared with all participants to explain the output from a 3D surface imaging device. A copy of their individual participant data was provided to any participant who requested it. Following the body measurement session, the second stage interview explored the same themes as in the first stage with the objective of exploring ways in which initial perceptions of participants were changed or confirmed by experiencing the different measurement procedures and any new perceptions or ideas that were generated.

Each participant's data was anonymised using a simple naming convention including participant ID, date created, etc. and encrypted in a Microsoft Excel file.

The interviews were voice-recorded and transcribed using Otter. ai transcription software [52].

### 2.3 Data analysis

The research team immersed themselves in the data by listening to the audio interviews and reading and editing the transcripts (AC) produced by Otter. ai.

The data was analysed by the research team (LN, MT, SM, SC) using the qualitative framework analysis approach [53,54]. This approach was developed in an applied research context to systematically manage qualitative data to identify potential for actionable outcomes by providing transparent results and conclusions that can be related to the original data.

### 2.4 Data synthesis

Qualitative analysis was undertaken using framework analysis [55]; Data was analysed manually in the following five stages.

I. familiarising (reading and rereading data transcripts)
II. identifying a thematic framework (the theory changes thematic framework informed the analysis)
III. indexing (entered short summaries into the coding frame)
IV. charting (entering themes into a matrix using columns and rows for summarised data)
V. mapping and interpretation (comparing data excerpts, searching for patterns, and seeking explanations for patterns in the data).

Following Gale et al. (2013) quotes were extracted from the transcripts to populate the framework [53].

The data is reported using a Standards for Reporting Qualitative Research (SRQR) using the consolidated criteria for reporting qualitative studies (COREQ), based on a 32-item checklist [54].

## 3 Results

### 3.1 Part 1: online questionnaire

After removal of duplicates and null responses, 49 respondents completed the initial online questionnaire. The demographic breakdown of respondents is shown in Table 2.

**Table 2:** Demographic breakdown of respondents to online questionnaire and interview.

**Sex – n (%)**	In questionnaire (n = 49)	In interviews (n = 26)
Male	18 (37 %)	13 (50 %)
Female	31 (63 %)	13 (50 %)
**Age (years) - n (%)**		
18-35	21 (43 %)	10 (38 %)
36-45	3 (6 %)	1 (4 %)
46-55	8 (16 %)	5 (19 %)
56-65	12 (25 %)	7 (27 %)
65+	5 (10 %)	3 (12 %)
**Race/Ethnicity – n (%)**		
Asian/Asian British - Bangladeshi	1 (2 %)	1 (4 %)
Asian/Asian British - Pakistani	1 (2 %)	0 (0 %)
Asian/Asian British - Indian	3 (6 %)	0 (0 %)
Mixed – White & Black African	1 (2 %)	1 (4 %)
Black/African/Caribbean/Black British - Caribbean	5 (10 %)	2 (8 %)
Other ethnic group - any other background	1 (2 %)	1 (4 %)
White- British	36 (74 %)	20 (77 %)
Asian/Asian British – Chinese	1 (2 %)	1 (4 %)
**BMI status**		
Underweight (<18.49 kg/m^2^)	–	0 (0 %)
Healthy Weight (18.50–24.99 kg/m^2^)	–	13 (50 %)
Overweight (25.00–29.99 kg/m^2^)	–	8 (31 %)
Obese (>30.00 kg/m^2^)	–	5 (19 %)

#### 3.1.1 Methods used to assess changes in body shape and size

**Fig. 2:**
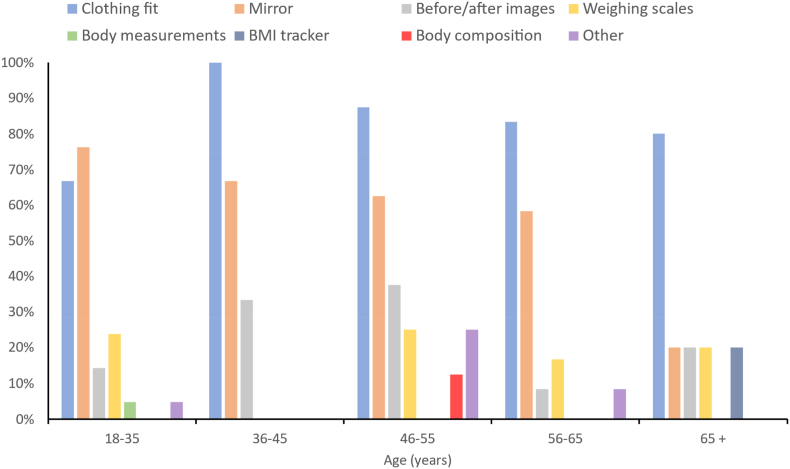
Proportion of respondents within different age categories that use different methods for assessing changes in body shape and size.

In the questionnaire, participants were asked how they usually assess for changes in their body shape and size. Across all age groups, the most common methods for assessing changes to body shape and size were how their clothes fit and looking at their reflections in the mirror (Fig. 2). Clothing fit was the primary method used to assess changes in body shape by participants aged over 36 years of age. In contrast, for participants in the youngest age group (18–25 years), looking in the mirror was the most common method (76 %), followed by clothing fit (60 %).

Nb: Body measurements = use of an anthropometric tape for girth or length measurement e.g., hip circumference; Before/after images = the use of photographs and images to monitor and measure changes in weight.

**Fig. 3:**
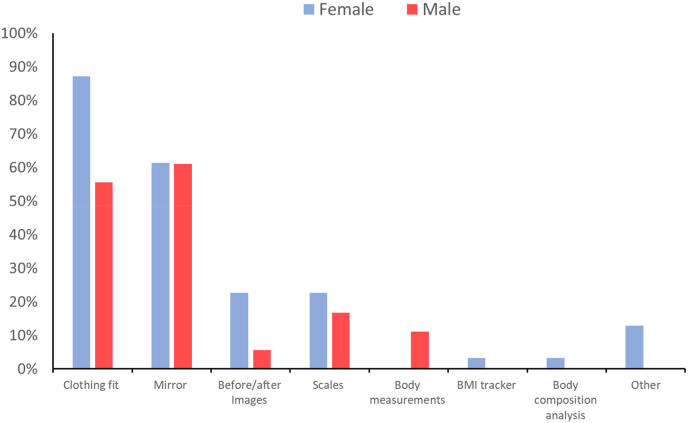
Proportions of males and females that use different methods for assessing changes in body shape.

For both male and female respondents, the most common methods for assessing changes in body shape were clothing fit and looking in the mirror (Fig. 3). Female respondents also mentioned using subjective measures such as mobility, walking speed and energy levels, as well as looking at before and after images. One female respondent, who disclosed additional health issues, also mentioned using a BMI tracker. None of the females reported using traditional body measures, such as waist girth. For the male respondents, the two most common methods for assessing changes in body shape were looking in the mirror and clothing fit. In contrast to the female respondents, 11 % of male respondents reported using body measures to assess changes in their body shape.

#### 3.1.2 Tools used by you or someone else to measure your body in the past

**Fig. 4:**
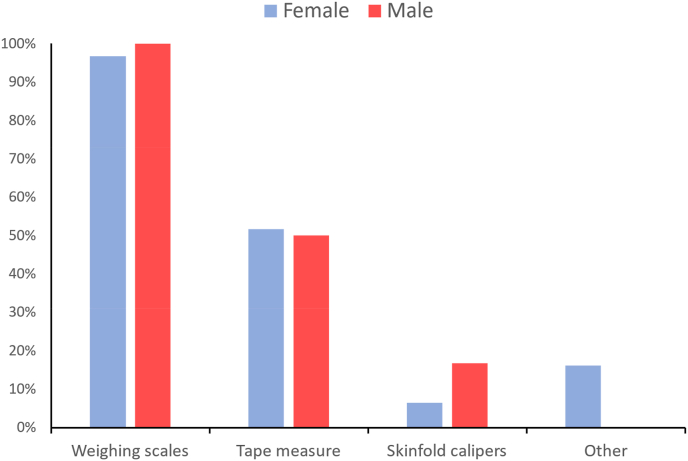
Proportions of males and females that have used/been measured using different body measurement tools.

The online questionnaire also asked participants what tools have they or someone else used to measure their body in the past. This could have included measures taken by medical practitioners, gym instructors or other health professionals. For both male and female respondents, the tool they reported as having been used most often by themselves or others to measure their body were weighing scales (Fig. 4). In addition, around 50 % of males and females both report that they have been measured using a tape measure by themselves or others previously. This is contrary to the methods they report using to assess changes in their body shape regularly themselves. There were no meaningful differences in tools used between different age or ethnic groups.

### 3.2 Part 2: semi-structured interviews and 3D surface imaging

In the second stage of this study, participants were selected through purposive sampling. Individuals who completed the online questionnaire, had consented to being contacted about further research, and who were not at risk from the BDD screen were invited to complete semi-structured interviews. In total, interviews were conducted with 26 individuals, with equal numbers of males and females as well as a good spread of ages, BMI values and ethnicities throughout the sample.

Three overarching themes were identified following thematic synthesis; (1) Technology; (2) Participant experience and (3) Perceived benefits and uses. Each theme was then divided into sub-categories and responses were analysed before and after experiencing the various measures of body morphology, as described in the methods. A summary of themes and sub-themes can be found in Table 3.

**Table 3:** Themes and sub-themes from qualitative interviews with example quotes (BMI= Body Mass Index; IMD= Index of Multiple Determinants of Deprivation).

Themes	Sub-themes	*Example quotes*
1. Technology	1.1 Translation of technology outputs into user friendly messaging	*“And if you're just measuring (data) for measuring's sake, no point. If, if the technology is designed to capture more information, so it can be used in terms of health care and monitoring your health going forward. It's got to be positive.” (P19 Male, Chinese,* 23 yrs*, BMI: 26.2, IMD: 3)* *“I find that quite difficult to interpret the figures. The image is very clear. But all those figures. I'm like, Ooh, I don't know.” (P16 Female, White British,* 64 yrs*, BMI: 34.4, IMD: 9)* *“You can see your own body shape … very, very obvious way. Tells the … like the parts in your body that usually you can't measure by traditional method.” (P19 Male, Chinese,* 23 yrs*, BMI: 26.2, IMD: 3)* *“Because it (the 3D surface imaging device) gives you so much information. The fact it gives you your arms, your legs, your neck … (…) whereas that (weighing scales, tape measure and stadiometer) gives me just a little bit of … a little bit of information, that gives me a lot of information.” (P22 Female, White British,* 52 yrs*, BMI: 41.9, IMD: 6)* *“You get, you know, the blue and red images is a good way, a more accurate way of doing it rather than just standing on a scale. It gives you ….you get an idea of places where you need to lose weight. And you also know when you're losing too much weight …..but it's a good way of doing it rather than standing on a scale.” (P15 Female, White British,* 60 yrs*, BMI: 31.9, IMD: 5)* *“I think it's just good for people to see visually certain areas of the body and see what they look like and maybe track progress over time.” (P1 Male, White British,* 28 yrs*, BMI: 24.7, IMD: 9)*
	1.2 Application, practicality and scepticism	*“I think anything that's non-invasive is good. No contact, no physical contact. I said before about that, you know - some people don't like to be touched ….especially by strangers.” P18 Male, White British,* 64 yrs*, BMI: 36.2, IMD: 1)* *“I was expecting it's just going to be some photos …..I didn't expect it to be so quick. I think that I was in the TARDIS.” (P14 Male, White British,* 28 yrs*, BMI: 24.7, IMD: 9)* *“I think it's important to have both. You need … you need that visual look, to bring it home to you that you are fat. But you also need the numbers to compare against your body mass and to also bring it home that you are overweight for your height. So, you need both really. It works side by side.” (P15 Female, White British,* 60 yrs*, BMI: 31.9, IMD: 5)*
	1.3 Novel technology	*“It's a completely new thing to me. So, I was excited to see the picture.” P2 Female, Other,* 24 yrs*, BMI: 25.3, IMD: 4* *“… it's not intrusive, and, and the data that you get from it is phenomenal.” P26 Female, White British,* 55 yrs*, BMI: 22.1, IMD: 2* *“It was a bit of a shock to see me there in 3D.” P15 Female, White British,* 60 yrs*, BMI: 31.9, IMD: 5* *“I look forward to seeing it in the hospital. If it ever gets that far!” (P18 Male, White British,* 64 yrs*, BMI: 36.2, IMD: 1)*
2. Participant Experience	2.1 Questioning the validity of the method	*“I guess waist is probably the most standard measure. Mine seems to vary considerably, depending on things like, what stage of my cycle I'm in and that sort of thing. I tend not to do it that often because … because of that, because of the fluctuations.” P9 Female, White British,* 35 yrs*, BMI: 26.7, IMD: 7* *“I don't believe it (the weighing scales) ….partly. Because I look at myself and I see, well it hasn't changed much. I may be wrong. Maybe there are changes going on there that I don't pick up. Maybe I should believe it a bit more, but …” P10 Male, White British,* 74 yrs*, BMI: 29.2, IMD: 5*
	2.2 Positive Experiences	*“Um, no, it wasn't scary. It was actually comfortable. Yeah.” P2 Female, Other,* 24 yrs*, BMI: 25.3, IMD: 4* *“And I think it's a good indication of kind of where you are (body weight), because I know that my weight should be maintained. So, I kind of know figure I'm looking for.” P6 Female, White British,* 24 yrs*, BMI: 24.9, IMD: 8* *“They're easy. They're quick. They're very accessible. You've just got to sort of stand on the set of scales, put the tape measure around your waist and look at that, so they're dead easy, they're quick and convenient. Easy to use.” P9 Female, White British,* 35 yrs*, BMI: 26.7, IMD: 7* *“I just notice that a lot of my clothes that I have - particularly suits for work and that - are all too big now because I've lost some weight and … my trousers, I used to always get 32 waist. Now I quite easily fit into a 30. Boring, but good.” P24 Male, White British,* 57 yrs*, BMI: 22.6, IMD: 5*
	2.3 Negative Experiences	*“And, different brands of clothes measure very, very differently. So you've got to think, I've put a size 10 and it will be equal to a size 12.” P26 Female, White British,* 55 yrs*, BMI: 22.1, IMD: 2* *“Because then I think you get into that body dysmorphia thing where you just overanalyse and self (assess too much than what is mentally healthy, I think.” P25 Male, White British,* 26 yrs*, BMI: 21.7, IMD: 5* *“I suppose it's the same thing as a scale. You could get to the point where you're trying to pinpoint tiny, micro, little changes in your body and, you know, oh, I want to improve my bust, or I want to get rid of my thighs, or I want to do that, and I suppose that level of getting obsessive with things.” P22 Female, White British,* 52 yrs*, BMI: 41.9, IMD: 6* *“It is a bit intrusive (manual measures) … especially when you know you're fat. It's like thinking, oh, you know, this is terrible, really. Because you can … you can feel the tape going around you, and you know it's going to be a big measurement. So, it's intrusive in that way.” P15 Female, White British,* 60 yrs*, BMI: 31.9, IMD: 5* *“I'm okay about it, but I can see not everybody would be comfortable with that because … especially when it comes to weight, people would be a bit uncomfortable, especially that you're touching them.” P17 Female, Black Caribbean,* 51 yrs*, BMI: 30.1, IMD: 2*
3. Perceived Benefits and Uses	3.1 What does the 3D surface image represent and what additional benefits it brings	*“Because if this equipment is in the gym, I think people they will see their body shape changing over time. Because it shows a direct image of myself. So then I'll see like which parts I need to more focus on.” P19 Male, Chinese,* 23 yrs*, BMI: 26.2, IMD: 93* *“It can show you as opposed rather than just looking in the mirror … how you're like … what your posture is (indistinct) for like postural control ….I know that my left shoulder is either higher or lower. (Indistinct) I got hit by a car.” P12 Male, White British,* 25 yrs*, BMI: 22.9, IMD: 8*
	3.2 Interest in knowledge and data generated	*“To have it there shown that these are the places where you are fat. And you've got to do something about it. It's like, almost like having a blood test, isn't it? You know, you've got the results and you're there, and it's staring at you.” P15 Female, White British,* 60 yrs*, BMI: 31.9, IMD: 5* *“I don't like looking at the image though. I REALLY don't like that picture of me. But then on the other hand, that's … that's how I look.” P16 Female, White British,* 64 yrs*, BMI: 34.4, IMD: 9*
	3.3 Motivational and emotional response to using body measurements	*“I was diagnosed with fatty liver. And it scared me a bit …...So I just think generally I might … I would benefit from some weight loss. But you know, when I … undress, I'm quite appalled at the way I look really. But, it should shock people into doing something.” P15 Female, White British,* 60 yrs*, BMI: 31.9, IMD: 5* *“I suppose my motivation are, I play football, bit of sports, so I like to be obviously fit and healthy for that. The other reason's purely for like aesthetics, just to obviously look a certain way ….like looking in the mirror in the morning or an afternoon after you've had a workout … seeing if you've made any progress … I sort of track how much I weigh every now and again ….I don't really put much importance into that really. it's more about the visual, rather than the actual reality, I suppose.” P1 Male, White British,* 28 yrs*, BMI: 24.7, IMD: 9* *“Yes, it would (help me) … because I'll be checking my body every time. How much … how much fat have I lost? Or am I putting it on? It (the scan) would be very, very helpful … in about two weeks or after (last GP appointment).” P14 Male, Asian-Bangladeshi,* 60 yrs*, BMI: 28.6, IMD: 1* *“I really like that ….information and then to compare ….in another sense, I'd worry I'd get too fixated on it ….You know, would I start to lose the will to live by looking at all of them ….what would be helpful for me is if I could just say, right, I only want to look at this, this and this ….don't give me my calf measurements.” P13 Female, White British,* 51 yrs*, BMI: 20.1, IMD: 10*

#### 3.2.1 Theme 1: Technology

The subject of technology was a consistent occurrence during the semi-structure interview, which is unsurprising given the fact that none of the participants had experienced the 3D surface imaging device before. Three distinct subcategories were identified during the discussion around technology, which are presented below.

##### 3.2.1.1 Translation of technology outputs into user friendly messaging

Participants were particularly focussed on the data generated from the 3D imaging scanner. They described how the data was both complex to understand initially, but has potential to be useful. Participants described how if the data was used and presented correctly with a meaningful association to health, then it would be considered to be a positive feature. However, some participants expressed concern that the amount of data provided by the 3D surface imaging device might cause them to be overwhelmed, particularly when people are used to simple height, weight and waist girth measurements.

Participants alluded to the additional precision and value of having a visual output of your body image, in addition to standard measures of assessing body size and reliance on self-assessment.

The ability to obtain site specific images and measures was commented on which was welcomed as useful information, and provided more context to weight loss and weight gain information which could be motivational.

The visual output of the 3D image added meaning for participants above “just numbers” and was considered beneficial for tracking changes over time.

Participants recognised that they could compare their 3D surface images at multiple timepoints to see how their body shape has changed. This feature has the potential to be beneficial for monitoring both aesthetics and changes in their health.

Overall, participants appeared accepting of the surface imaging device and found that it was able to provide useful information which could be generated into user-friendly outputs for both short- and long-term use. However, the vast quantity of data that was provided could be overwhelming, and unnecessary for many and would need to be selected specifically to meet the needs of individuals and to ensure it was presented in a way which was meaningful to the users.

##### 3.2.1.2 Application, practicality and scepticism

Participants described how 3D surface imaging seemed a much less intrusive way of collecting body measures, compared to manual measurements techniques, and felt that it was a more private way of being measured which they commented positively on.

Participants described how data capture using the 3D surface imaging device was surprisingly quick, especially for the quantity of data that was collected in comparison to the manual measures, and as a result felt that this could be done by themselves in a GP or leisure centre.

However, participants felt that the 3D surface imaging device would need to be used in conjunction with other measures so that body shape could be compared with more familiar outputs such as BMI. They felt that the main drawback of the 3D surface imaging device was the lack of body composition data which was important to their understanding of health.

Additionally, the participants were quite insightful about the practicalities of the current kit, which is expensive and takes up a lot of space. They felt that there were some benefits to this such as the possibility of it being easily adapted for wheelchair users but there was an awareness of how the size and cost would not be embedded easily into the current healthcare or primary care environment and would therefore be of limited use.

##### 3.2.1.3 Novel technology

Initially, participants were apprehensive and excited about the use of a new technology, and felt that the 3D surface imaging device was quick, efficient, and accurate.

Participants reported that they felt comfortable using the equipment and that this was beneficial, particularly for them to look at trends in their body shape and changes over time.

Participants also noted that the surface imaging device was less intrusive than ‘hands-on’ anthropometry techniques such as waist circumference, as a tool for measuring body shape and size and that it was able to generate large quantities of specific information which accurately described them.

Whilst people liked the perceived accuracy of the data that was presented and most felt comfortable with the 3D surface image that they were shown, there was some reports of shock and/or surprise with the output and seeing their bodies from a completely different perspective.

It was also apparent that the description of the process given to individuals prior to using the 3D surface imaging device was incomplete as participants reported that they expected to be scanned for a longer duration (up to half an hour), that they expected a ‘photobooth’ countdown and flashing lights or postural adjustments.

Some participants felt that the 3D surface imaging device should be rolled out immediately, *IMD,* with additional scaled up trials and deployment for public and professional use.

#### 3.2.2 Theme 2: Participant experience

Theme two explored the participant experience in relation to previous use of methods to determine body weight and shape. Sub-themes that emerged from this discussion were.

##### 3.2.2.1 Questioning of validity and applicability

Several participants were sceptical about the use of traditional methods of assessing body morphology and how applicable the results were to them personally. One main factor was the high degree of fluctuation between monitoring sessions and how this reduced the impact of the result on the individual. These fluctuations caused some participants to use traditional measures less frequently, or with less confidence as a result.

Participants felt that traditional measures of body morphology such as weight and waist circumference over- or under-measured changes in their bodies leading to a sense of disbelief or mistrust in the measurements.

Mistrust of the accuracy of data produced by traditional body measures appeared to be a common theme throughout the qualitative sessions.

##### 3.2.2.2 Positives of body morphology measures

There were several consistent positive themes relating to general body morphology measures. The most common being that participants felt that using measures of body morphology permitted the ability to notice change (particularly in body weight) and to assess if they were “on track”.

A number of participants employed the use of body morphology measures due to the speed and ease at which the measures could be taken, allowing the simple and rapid assessment of current weight status.

A third common topic was in relation to using existing clothing as a marker of body size. Additionally, the use of clothing, and how well the garment fit, was described as a measure of success or a motivational driver to facilitate a change in their behaviour.

Where participants were used to monitoring their body size and shape using both subjective and objective measures, they reported that these helped to assess whether they were ‘on track’ and provided a way of motivating themselves to take part in weight loss or gain strategies to maintain their weight.

##### 3.2.2.3 Negatives of body morphology measures

Using clothing to assess body size was a technique utilised by many participants. Though some participants had positive experiences of this method, other discussed their own negative encounters. Inconsistencies in garment sizing between different shops and brands results in lack of confidence in using clothing as a measure of body shape.

Some participants discussed their concerns related to varying measures of body morphology, with relation to the wider impact on an individual's wellbeing. A number of participants expressed concerns that the consistent use of body morphology measures could lead to individuals becoming obsessed with their own size and over-analysing the information generated, which could negatively impact the perception of oneself.

Some of these negative concerns were particularly related to the results obtained and use of the 3D surface imaging device.

After experiencing each of the manual measures of body size and shape, a number of concerns were expressed about the discomfort felt personally, or what others might experience. This was particularly evident for measurements of waist circumference using an anthropometric tape which were described as intrusive.

Other participants reported that being touched physically also had the potential to make them or others feel uncomfortable.

It was evident from the participants’ discussions that both the experience of having the body morphology measures taken, and the manner in which results were presented or perceived were causes of potential discomfort, even in a study population who had volunteered to have their body measurements taken.

#### 3.2.3 Theme 3: Perceived benefits and uses

##### 3.2.3.1 What does the 3D surface image represent and what additional benefits it brings?

Several participants saw the benefits of being able to gain a more in-depth view of their physique and being able to view it from different perspectives, as opposed to just seeing a weight value. Some participants seemed to have a perception that because the 3D surface imaging device enabled them to see where their body mass was distributed and they could see where they had greater amounts of mass, that they then might be able to target specific areas on their body to lose weight through diet or exercise.

As an adjunct to weight monitoring, participants identified other potential benefits of using the 3D surface imaging device, such as changes in posture.

This type of information could potentially have uses within rehabilitation or physiotherapy applications for those living with obesity, where muscular imbalances have been identified but are not perceptible to the patient. These body images could be shown to the patient to help them visualise these issues and then monitor improvements over time following remedial physical therapy.

##### 3.2.3.2 Interest in knowledge and data generated

Several participants seemed to have a genuine interest in the data generated by the 3D surface imaging device and how it could be used, this appeared to correlate primarily with individuals who described an interest in quantitative data. These individuals commented on how they liked the objective nature of the data produced by the 3D surface imaging device, likening it to having a blood test. For these individuals, this data provided an objective representation of what their body looks like, which they couldn't avoid.

Though some participants commented that they didn't like seeing the images, they recognised that it at least gave them an objective measure of their body shape, which they could then use as a starting point for them to then make changes.

However, it is hard to determine from this study, whether people would be able to use the data objectively to make appropriate lifestyle changes in order for them to achieve their weight goals, or whether the emotional response to seeing the images would override the ability to interpret and process the available data.

##### 3.2.3.3 Motivational and emotional response to using body measurements

Several participants discussed health concerns being a primary motivator for measuring their body, either because of health issues that they have previously had themselves, or health issues that other members of their family have suffered from, which they could potentially also be at risk of.

Other primary motivations for participants collecting body measurements included staying healthy to enable them taking part in physical activity and sport, as well as for purely aesthetic reasons.

Participants felt that if they could track visual changes in their appearance following periods of exercise, either by seeing how their body shape itself had changed, or by simply whether their clothes fit them well, then they are making good progress rather than whether their weight has changed.

There were quite disparate emotional responses to taking body measurements, ranging from a quite pragmatic understanding that they have a purpose within health monitoring, to the potential damaging effects of fixating on body image. Some participants questioned whether having access to improved measures of body shape or the ability to see oneself in a detailed 3D surface image might contribute to a perceived need to conform to accepted societal norms of body shape, as well as the risk of body image-related disorders, such as body dysmorphia.

Whilst it is positive that people felt that the 3D surface imaging device would be motivational if used appropriately, it would also be important to understand those at risk of becoming, or those being obsessive, with taking body measurements and have a system in place which prevents and addresses this behaviour.

## 4 Discussion

As far as we are aware, this is the first study which provides insight into why individuals use body morphology and body measurement techniques, which measurements are preferred and perspectives of novel 3D surface imaging techniques.

Previous research has suggested that having alternative indices of success (besides weight), which increase self-efficacy and perceived autonomy around weight management, could help avoid the failings of short-term weight loss goals by embedding longer term indicators of success which are acceptable to participants [56]. In the current study, participants measured changes in their body shape and size by how their clothes fit, looking in the mirror and using weighing scales, although there were some gender differences with females also comparing before and after photographs of themselves, and using more subjective measures of health such as perceived fitness levels. However, previous research suggests that measures such as clothing fit, and size can be variable, unreliable and may lead to altered body dissatisfaction and confidence [57].

Recent evidence suggests that self-monitoring of body weight is associated with successful weight-loss outcomes [58,59] and is a popular practice within many weight-management interventions [60]. Given the potential benefits related to improving the effectiveness of weight-loss interventions, little evidence exists alluding to the habitual frequency of self-weighing, or other self-applied techniques of assessing body morphology.

In addition to the lack of evidence of regular self-monitoring of body size, there is little evidence of why people might self-monitor in the first instance. In the current study, though almost all of the participants reported that they have had their body weight monitored by someone else in the past, only ∼15–20 % of participants regularly use weighing scales themselves, with clothing fit and mirror image being the most used techniques. Chambers and Swanson (2012) stated that self-weighing allows people to take appropriate action based on fluctuations in their body weight and is thus an effective self-monitoring technique [61]. However, despite evidence of minimal harmful psychological effects of regular self-weighing [62], a potential negative impact identified in the analysis relating to all measures of body size was on body perception. Participants in the current study opted for more visual and perceptual measures of their body, with some attributing this to aesthetic reasons, although previous research suggests that mirror-based observations of size are of variable accuracy [63]. However, participants felt that perceptual changes derived from clothing fit and reflection in the mirror, were a better indicator of their progress than weight on a scale. Other primary motivations for participants collecting body measurements included staying healthy to enable them taking part in physical activity and sport. However, several participants discussed health concerns being a primary motivator for measuring their body, either because of health issues that they have previously had themselves, or health issues that other members of their family have suffered from, which they could potentially also be at risk of. This is supported by prior research which demonstrates a beneficial effect of self-monitoring of health metrics in weight management [64]. Therefore, people may benefit from being able to self-monitor potential indicators of health risks (i.e., waist circumference, fat %) via the use of novel technologies such as 3D surface imaging, rather than waiting for their healthcare provider to identify issues. This ability to self-measure body morphology to a higher degree of accuracy and granularity could also perhaps provide individuals with a sense of empowerment in being able to take charge of their own health. This data provides evidence that the motivations behind self-monitoring of body size range from increasing or maintaining physical capability, improving or maintaining health and attaining favourable aesthetic results.

BMI and waist circumference measures are widely used in the UK and recommended by NICE weight management guidelines [65]. Participants were familiar with standard body morphology measurements such as height, weight, waist circumference and BMI and still felt that these measures were valuable to them and their understanding of health. However, when participants were asked in the online questionnaire what methods they used to assess changes in their body shape and size, only a small proportion of individuals reported using these traditional body measures regularly themselves, preferring instead to use more visual or subjective measures. Whilst traditional measures, such as BMI, are widespread and familiar to the UK population, they have been heavily criticised as they do not reflect the adiposity or lean tissue composition of an individual and therefore poorly correlate to health [66]. Despite over 50 % of respondents in part 1 of the study stating that body measurements (girth or circumference measured by an anthropometric tape) had been employed by either themselves or by others in the past, only 11 % of male respondents and no female respondents reported using this method regularly. When this topic was explored in part 2 of the study, it became apparent that some participants found the use of the anthropometric tape to be uncomfortable and intrusive. The use of waist circumference (WC) as a measure of body size has been previously examined from both a practitioner and patient perspective. Interestingly, though few barriers were raised from the patients with regards to the use of WC, the perspective of the practitioners was that some patients might feel embarrassed about the measure, and that it was a time-consuming process [67]. There appears to be a possibility that a combination of the practitioners’ reluctance to conduct WC measurements, coupled with the negative perceptions of WC measurements expressed in the current study, might be a reason why so few participants reported using body circumference to track changes in body shape and size. However, despite their familiarity with traditional methods, participants felt that the 3D images provided more accurate, objective information which was more site-specific, and that this data was a useful adjunct to more traditional measures.

The main reported benefit of the 3D surface imaging device was that it was perceived to provide a large volume of accurate information, quickly and in a non-intrusive manner. Given that several participants expressed concerns over the validity and applicability of traditional methods of measuring body shape and size, this element of accuracy is of particular significance. However, participants perceived that the 3D surface imaging data was more accurate than traditional methods, despite having no evidence to validate this. Some research has shown that whilst 3D surface imaging devices have great replicability of results, and remove the human error associated with traditional body measurements, they may overestimate waist and thigh circumference, particularly in those with smaller thigh circumferences [68]. Whilst the quantity of information had value for some, it was considered overwhelming for others. This then suggests that further work would be necessary to identify the key information from the imaging device and how to present this in the most useful format so that individuals can understand and use data which is meaningful to them in an actionable way. This process of meaning-making from data has been categorised into 3 areas-supporting digital data practices, interpretation and contextualisation, and inclusion and interaction [69]. This would also involve additional training of healthcare professionals or leisure centre staff where 3D surface imaging services are available.

The speed of 3D surface imaging, a common positive experience reported by participants, as opposed to the manual measures is beneficial and may help to increase engagement with attendance at appointments, or frequency at which HCPs are able to take measurements. A criticism of manual measures is that they are time-consuming, especially when multiple measurements are required [70] so the 3D surface imaging device offers a distinct advantage in terms of the amount of time to collect measures.

Perhaps most importantly, the non-intrusive manner in which the 3D surface imaging device collects data could be of most benefit, as several participants expressed negative experiences when waist circumference was measured, as observed in previous literature [11]. By providing a 3D surface image as an alternative measure of body morphology, especially for those patients who are deterred from attending clinical appointments due to the current invasive methods, 3D surface imaging could improve uptake of appointments and provide a more inclusive, low-stigma environment [10,11]. Also, there was a sense that the ability to measure body morphology to a higher degree of granularity could also perhaps provide individuals with a sense of empowerment in being able to take charge of their own health. In contrast, some participants expressed concerns about potential damaging effects of fixating on body image and targeting weight loss from specific locations, and that having the ability to see oneself in a detailed 3D surface image might contribute to a perceived need to conform to accepted societal norms of body shape, which could put people at risk of body image-related disorders, such as body dysmorphia [49]. However, there is insufficient evidence to support targeting weight loss from specific locations on the body [71,72], so this might give users unattainable goals, if they are focused on changing the shape of a specific part of their body, which could cause them to become demotivated when that aspect of their shape doesn't change as they hope.

Previously, healthcare professionals have been criticised for inadequate communication skills, lack of training and use of stigmatising language when performing body measurement 3D surface imaging is an accepted methodology and may reduce anxiety in assessments [73] which may also contribute to the negative perceptions of body morphology measures. The use of people-first language and effective communication of information is vital irrespective of the method used to capture body measurements, Therefore, training will need to be delivered with practitioners prior to the adoption of any new technologies, including disseminating the outputs of the 3D surface imaging device with individuals in a people-first, inclusive language which is actionable for individuals [74].

### 4.1 Strengths and limitations

As far as we are aware, this is the first study which assesses the perspectives of a diverse group of individuals on the experience and use of 3D surface imaging technology as an alternative or adjunct to traditional body morphology measurements. The study recruited a diverse range of males and females from different ethnic backgrounds, areas of deprivation and age groups, representative of the local population. The study assessed acceptability of the technology and participants suggested additional potential uses of the 3D surface imaging device to consider in future work.

At present, the large range of available hardware, software, calibration techniques, anthropometric definitions, and data collection procedures used by different 3D imaging systems, makes the comparison of body composition estimates from these devices difficult, thereby limiting its suitability for use in research and practice. Whilst 3D imaging can provide some data regarding fat distribution, it is not a direct measure of body composition, nor is it specifically validated as a predictor of cardiometabolic outcomes. A further critical review of 3D surfacing imaging as a method of estimating body composition is presented by Heymsfield et al. [34], with further research suggested in this article required to support the use of 3D imaging in clinical practice.

However, this is still a relatively small sample size due to the exploratory nature of this initial study and further research is required to fully understand the psychological implications of using this type of technology within healthcare or commercial settings in a larger population group. 3D imaging is not a recommendation in obesity management recommendations for clinicians or practitioners.

### 4.2 Recommendations for practice

There is a need to recognise that traditional body measurements and body morphology can feel intrusive, awkward and stigmatising for some individuals and that in some cases, this can prevent or delay access to healthcare. Body measurements should always be done in discussion with the individual, in an appropriate setting and only when deemed clinically relevant or necessary. For individuals who feel uncomfortable with traditional measures and methods, alternative solutions should be considered. 3D surface imaging may be a suitable alternative method for collecting body measures for some individuals, with detailed measures able to be collected quickly and without requiring physical contact from a healthcare professional or other practitioner. However, further training on non-stigmatising, people-first language and how to share 3D surface imaging information in a meaningful way should be prioritised.

### 4.3 Future research

Future research should investigate the use of low-cost tools to acquire shape anthropometrics from the surface of the body, enabling all practitioners to assess human morphology without relying on expensive 3D surface imaging devices. Most 3D surface imaging devices are large pieces of equipment which are generally static, and though some devices have translated from fixed laboratory instruments into commercially available portable devices, they currently cost significantly more than traditional weighing scales and body measurement tools frequently used in practice. However, more portable body morphology measurement tools are being developed. These low-cost tools could either be physical objects, allowing practitioners to manually measure body shape features such as curvature and simple ratios, or 3D imaging apps which can be embedded into lightweight devices such as smartphones or tablets and are able to replicate the outputs of existing 3D surface imaging systems and capture shape anthropometrics to a required level of accuracy.

In addition, if 3D imaging technologies are to be of benefit to population health, there needs to be assessment to understand whether these technologies have a specific, long-term benefit to weight and obesity management in comparison to traditional anthropometry techniques.

In conjunction with this, people with lived experience of being measured in a 3D surface imaging device should be involved in co-developing a description of the procedure to provide to patients prior to their appointment to reduce any anxiety and set expectations. Whilst this paper initiates discussion regarding individuals’ perceptions of body contour and shape, and how they might use this data, this would be worthy of further research.

Additional research is required to assess how the technology can be used at scale, and the training and resource needs required for successful implementation in public health and wellbeing environments. The impact of the availability of this technology to weight management also warrants further investigation.

## 5 Conclusion

The study aimed to understand acceptability of traditional and 3D surface imaging-based anthropometric measures.

• Traditional methods of assessing body shape and size, such as weighing scales and circumference measures, were found to be seldom used in comparison to more subjective, aesthetic measures, such as reflection in the mirror or clothing fit.
• The novel 3D surface imaging device was widely accepted by participants and was perceived to have some benefits over traditional anthropometric measures such as being less intrusive, quick, accurate and able to produce detailed additional information to assess body shape changes over time.
• However, data produced by the 3D surface imaging device was recognised to be complex, overwhelming and would require effective training and communication strategies for healthcare professionals and service users for the results to provide useful health metrics as an adjunct to traditional anthropometric techniques.

## Author contribution

The concept of the submission was by LN, MT, SM and SC. Methodology development was done by LN, MT, SM and SC. Data curation was done by MT, SM and AC. Formal analysis was done by LN, MT, SM, SC and AC. Funding acquisition was done by LN, MT, SM and SC. The original draft was prepared by LN, MT and SM. The final draft of the manuscript was reviewed, edited, and approved by LN, MT, SM, SC and AC.

## Ethical review

All procedures were approved by the University Research Ethics Committee Sheffield Hallam University, reference number ER41719941.

## Source of funding

This work was supported by an internal University Fieldwork Funding grant, 10.13039/100010035Sheffield Hallam University.

## Declaration of artificial intelligence (AI) and AI-assisted technologies

During the preparation of this work the authors did not use AI.

## Declaration of competing interest

The authors declare that they have no known competing financial interests or personal relationships that could have appeared to influence the work reported in this paper.

## Appendix A Supplementary data

Questionnaire-the online questionnaire which collated the demographic data and screened for BDD.

Semi-structured interview guide-the interview guide which was used during the qualitative interviews.
