# Supplementary material for: Patient perceptions of three-dimensional (3D) surface imaging technology and traditional methods used to assess anthropometry
Source: Obes Pillars. 2024 Feb 1;9:100100. doi: 10.1016/j.obpill.2024.100100 (PMC10865393; doi:10.1016/j.obpill.2024.100100)
Supplement: Multimedia component 1 [file mmc1.docx]

Weight Management Strategies Focus Group - Baseline Questionnaire

Start of Block: Default Question Block

Q2

 **Patient and Public Involvement - Baseline Questionnaire
 Project Title: Exploring people’s perceptions of body measurement tool used in weight management.**

 **What is this project about?**You are invited to take part in a patient and public involvement (PPI) activity aimed at improving our understanding about perceptions of existing body measurement tools used in weight management and whether novel body measurement procedures could be a more appropriate alternative for underserved individuals.

 This baseline questionnaire will ask for some basic demographic information and ask for your thoughts on existing body measurement practices. In addition, if you are interested you will be invited to take part in stage 2, which will take the form of individual interviews conducted at the Advanced Wellbeing Research Centre (AWRC) in Sheffield. The interviews will further explore themes arising from the initial questionnaire. We will further expand on the idea of ‘future technologies’ that could be used in weight management or as a tool to assess personal development. 

 **What will happen to the collected information when this study is over?**Any information you provide in this questionnaire will be stored securely, and your identity/information will be kept strictly confidential. Only researchers in the research team (listed below) will be able to access your identity and information provided in this questionnaire. 
 In addition, you will be given the opportunity to continue your involvement beyond this PPI and be included as part of the project team/steering group for any grant applications that are submitted as a consequence of this specific PPI work - and costed in/reimbursed in accordance with the national guidelines. If you are included as part of the project team/steering group, you could be asked to contribute to carrying out research projects, as well as the dissemination and implementation of research findings.

 **Can I tell other people about this questionnaire and getting involved?**Yes of course. We are interested in having involvement from individuals, aged 18 years and above, from as diverse a group as possible. Therefore, if you are aware of, or in touch with local/national community groups that might be interested in being involved feel free to pass on details of this questionnaire to them.

 **What if I would like further information about the study?**If you would like to know more at any stage, please feel free to contact the researchers named at the bottom of this page. 

 **The legal basis for research for studies.**The University undertakes research as part of its function for the community under its legal status. Data protection allows us to use personal data for research with appropriate safeguards in place under the legal basis of public tasks that are in the public interest. A full statement of your rights can be found at https://www.shu.ac.uk/about-this-website/privacy-policy/privacy-notices/privacy-notice-for-research. However, all University research is reviewed to ensure that participants are treated appropriately and their rights respected. This study was approved by the University Research Ethics Committee - Converis number ER41719941. Further information at https://www.shu.ac.uk/research/ethics-integrity-and-practice

| Page Break |  |
| --- | --- |

Q31 I confirm that I have read the participant information sheet for this study and am happy to complete this baseline questionnaire.

- Yes (1)
- No (2)

Skip To: End of Survey If I confirm that I have read the participant information sheet for this study and am happy to compl... = No

| Page Break |  |
| --- | --- |

Q1 Today's date:

________________________________________________________________

| 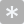 |
| --- |

Q2 Name:

________________________________________________________________

End of Block: Default Question Block

Start of Block: Block 1

Q1
What gender do you identify with? *Please tick one of the options below*.

- Male (1)
- Female (2)
- Other (3)
- Prefer not to say (4)

| Page Break |  |
| --- | --- |

Q2 What is your age in years?

________________________________________________________________

| Page Break |  |
| --- | --- |

Q5 What is your ethnic background? Please select one:

▼ White - English / Welsh / Scottish / Northern Irish / British (1) ... Other ethnic group - any other background (18)

| Page Break |  |
| --- | --- |

Q7 What is your postcode?

________________________________________________________________

End of Block: Block 1

Start of Block: General questions about body measurement

Q26 Are you actively trying to lose/gain/maintain weight at the moment?

- Yes (1)
- No (2)

Display This Question:

If Are you actively trying to lose/gain/maintain weight at the moment? = Yes

Q15 What are some of your key motivations for changing/maintaining your weight?

________________________________________________________________

| Page Break |  |
| --- | --- |

Q11 How do you usually assess for changes in your body shape and size?
 Examples may include looking in the mirror, clothes size/fit, flexibility, physical measures or any of the above.

- Before/after images (1)
- How well your clothes fit (2)
- Looking in the mirror (3)
- Other (please describe) (4) __________________________________________________

| Page Break |  |
| --- | --- |

Q10 What tools have you or someone else used to measure your body in the past?

- Weighing scales (1)
- Tape measure (2)
- Skinfold callipers (3)
- Other (please describe) (4) __________________________________________________

| Page Break |  |
| --- | --- |

Q12 We are currently investigating the use of 3D imaging technology in weight management strategies. This is a non-contact measurement device, which is painless and harmless, and involves the following: standing in a photo booth in form-fitting clothing for around 20 seconds seeing images of yourself from multiple angles potentially seeing aspects of your body coloured according to shape/size   
Do you consent to being contacted about taking part in the second stage of this study, which will explore your thoughts on the use of this technology in weight management?

- Yes (1)
- No (2)

Skip To: End of Survey If We are currently investigating the use of 3D imaging technology in weight management strategies.... = No

Display This Question:

If We are currently investigating the use of 3D imaging technology in weight management strategies.... = Yes

Q36 Are you able to travel to the Advanced Wellbeing Research Centre to take part in the second stage of this study? The address is: Olympic Legacy Park 2 Old Hall Rd, S9 3TY

- Yes (1)
- No (2)

Display This Question:

If Are you able to travel to the Advanced Wellbeing Research Centre to take part in the second stage... = Yes

Q32 Please enter your phone number in the space below.

________________________________________________________________

Display This Question:

If Are you able to travel to the Advanced Wellbeing Research Centre to take part in the second stage... = Yes

Q33 Please enter your email address in the space below.

________________________________________________________________

End of Block: General questions about body measurement

Start of Block: Body dysmorphic disorder foundation questionnaire

| 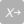 |
| --- |

Q16 How often do you do you deliberately check your feature(s)? Not accidentally catch sight of it. Please include looking at your feature in a mirror or other reflective surfaces like a shop window or looking at it directly or feeling it with your fingers.
 Choose the option that is closest to the approximate value.

- About 40 times or more a day (8)
- About 20 times or more a day (6)
- About 10 times or more a day (4)
- About 5 times or more a day (2)
- Never check (0)

| Page Break |  |
| --- | --- |

| 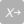 |
| --- |

Q17 To what extent do you feel your feature(s) are currently ugly, unattractive or ‘not right’?
 Choose the option that is closest to how you feel.

- Very ugly or "not right" (8)
- Markedly unattractive (6)
- Moderately unattractive (4)
- Slightly unattractive (2)
- Not at all unattractive (0)

| Page Break |  |
| --- | --- |

| 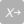 |
| --- |

Q18 To what extent does your feature(s) currently cause you a lot of distress?
 Choose the option that is closest to how you feel.

- Not at all distressing (0)
- Slightly distressing (2)
- Moderately distressing (4)
- Markedly distressing (6)
- Extremely distressing (8)

| Page Break |  |
| --- | --- |

| 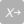 |
| --- |

Q19 How often does your feature(s) currently lead you to avoid situations or activities?
 Choose the option that is closest to the approximate value.

- Always avoid (8)
- Avoid about 3/4 of the time (6)
- Avoid about half of the time (4)
- Avoid about 1/4 of the time (2)
- Never avoid (0)

| Page Break |  |
| --- | --- |

| 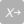 |
| --- |

Q20 To what extent does your feature(s) currently preoccupy you? That is, you think about it a lot and it is hard to stop thinking about it?
 Choose the option that is closest to how you feel.

- Not at all preoccupied (0)
- Slightly preoccupied (2)
- Moderately preoccupied (4)
- Very preoccupied (6)
- Extremely preoccupied (8)

| Page Break |  |
| --- | --- |

| 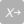 |
| --- |

Q21 If you have a partner, to what extent does your feature(s) currently have an effect on your relationship with them? (e.g. affectionate feelings, number of arguments, enjoying activities together). If you do not have a partner, to what extent does your feature(s) currently have an effect on dating or developing a relationship? If you are not actively looking to date or develop a relationship please select N/A.
 Choose the option that is closest to how you feel.

- Not at all (0)
- Slightly (2)
- Moderately (4)
- Markedly (6)
- Extremely (8)
- N/A (9)

| Page Break |  |
| --- | --- |

| 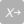 |
| --- |

Q22 To what extent does your feature(s) currently interfere with your ability to work or study, or your role as a homemaker? (Please rate this even if you are not working or studying: we are interested in your ability to work or study.)
 Choose the option that is closest to how you feel, then click on the 'Next' button below.

- Not at all (0)
- Slightly (2)
- Moderately (4)
- Markedly (6)
- Very seriously - I can't work (8)

| Page Break |  |
| --- | --- |

| 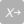 |
| --- |

Q23 To what extent does your feature(s) currently interfere with your social life? (with other people, e.g. parties, pubs, clubs, outings, visits, home entertainment).
 Choose the option that is closest to how you feel, then click on the 'Next' button below.

- Not at all (0)
- Slightly (2)
- Moderately (4)
- Markedly (6)
- Very severely (8)

End of Block: Body dysmorphic disorder foundation questionnaire

Start of Block: Block 4

| 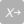 |
| --- |

Q24 To what extent, do you feel your appearance is the most important aspect of who you are?
 This is the last question. Choose the option that is closest to how you feel, then click on the 'Submit' button below.

- Not at all (0)
- Slightly (2)
- Moderately (4)
- Mostly (6)
- Totally (8)

| Page Break |  |
| --- | --- |

Q35 Do you consent for the information captured in this survey, once anonymised, to be used for related research purposes?

- Yes (1)
- No (2)

End of Block: Block 4
