# Supplementary material for: Patient perceptions of three-dimensional (3D) surface imaging technology and traditional methods used to assess anthropometry
Source: Obes Pillars. 2024 Feb 1;9:100100. doi: 10.1016/j.obpill.2024.100100 (PMC10865393; doi:10.1016/j.obpill.2024.100100)
Supplement: Multimedia component 2 [file mmc2.docx]

**Project Title: Exploring people’s perceptions of body measurement tool used in weight management.**

**Provisional Question schedule semi-structured topic guide**

**Part 1-PRE** (to be administered face-to-face prior to the body measurements)

Background

1. According to the answers you gave us in the initial questionnaire, you said that you were actively trying to [gain/lose/maintain] your weight. Can you tell you tell me a bit more about your motivation and how you measure whether you are being successful or not?

Prompts:

- Current situation
- Alone or with support?
- How are things going right now?

Methods for assessing changes in body shape and size

Usual Methods Used By Participant

1. You indicate that you use / have used _____________ for assessing changes to your body shape and size. Could you tell me how you feel about using this method?

Prompts:

- Convenience, ease and speed of use, cost…
- Motivation (feel of clothes, body profile…)
- Positives and negatives

Equipment & Tools Used

1. You indicate that you have had your measurements taken using the following equipment / tools. Could you tell me how you feel about this method(s)?

Prompts:

- Who administered? (self, health professional, friend, staff, weight loss group staff…)
- Preferred method(s) and reasons
- What method(s) don’t you like and reason(s)
- What makes a difference to your feelings (role of person - medical versus non-medical; gender; physical contact; personal space; discomfort, embarrassment, undressing, location, accessibility, duration, timing)
- Good experiences – what it made it so?
- Negative experiences – what made it so?

1. Do you currently use the measurements to track progress or to motivate you on your journey?

Perceptions and thoughts around scanning_

Read the following description of 3D body scanning:

“In a few seconds, our body scanner will take 20 3D photographs of your body from many different angles. If you look inside the scanner you’ll be able to lots of circular holes in its frame – this is where the cameras are positioned. When we take a scan, all the different 3D images are stitched together to make a single, full body scan of your body. We can take measurements from this scan to see how the size of the body changes over time. Or, if we have two scans taken at different times, we can compare them to see how the shape of the body has changed.”

1. What are your perceptions and thoughts of 3D scanning?

Prompts:

- Do you think it would be a useful measure for you?
- What do you think the positives and negatives would be about this measure?
- How do these differ from your current methods of measurement?
- Do you think this would help you to track your progress or increase your motivation to adhere to your body change plan?

**Take the following measurements :**

**Height (using stadiometer)**

**Weight (using scales)**

**Waist circumference (using tape measurement)**

**Ask participant to step into scanner**

**Part 2-POST** (to be administered face-to-face after the body measurements)

1. What were your thoughts and perceptions of all the measures we took today e.g. height, weight, waist measurement…
2. Now that you’ve had your 3D body scan, what were your thoughts and perceptions of this as a measure?

Prompts:

- How did it differ to your initial perceptions? Anything better/worse?
- Is there anything that you wish we had told you prior to the measurement that you wish you’d have known?
